# Virtual screening of immunomodulatory medicinal compounds as promising anti-SARS-COV-2 inhibitors

**DOI:** 10.2217/fvl-2020-0079

**Published:** 2020-05-21

**Authors:** Saad Salman, Fahad H Shah, Jawaria Idrees, Fariha Idrees, Shreya Velagala, Johar Ali, Abid A Khan

**Affiliations:** 1 ^1^The University of Lahore, Islamabad Campus, Islamabad, Punjab 44790, Pakistan; 2 ^2^Centre of Biotechnology & Microbiology, University of Peshawar, Khyber Pakhtunkhwa 25120, Pakistan; 3 ^3^Islamia College University, Peshawar 25120, Pakistan; 4 ^4^The Quarry Lane School, Dublin, California 94568, USA; 5 ^5^Department of Computer Science, Sukkur IBA University, Sukkur, Sindh 65200, Pakistan; 6 ^6^Center for Genomics Science, Rehman Medical Institute Hayatabad, Khyber Pakhtunkhwa 25100, Pakistan; 7 ^7^Muhammad College of Medicine, Peshawar 25000, Pakistan

**Keywords:** acute toxicity, drug repurposing, molecular docking, pharmacokinetics, severe acute respiratory syndrome-2

## Abstract

**Aim:** Severe acute respiratory syndrome coronavirus-2 (SARS-COV-2), a pernicious viral disease, causes acute respiratory distress responsible for mortality and morbidity worldwide. To screen different immunomodulatory medicinal compounds to unravel their interaction with SARS-COV-2 viral proteins. **Materials & methods:** A library of immunomodulatory medicinal compounds with antiviral capability were analyzed against SARS proteases, spike protein and nonstructural proteins (NSP-9, 15) using Autodock vina. **Results:** Out of more than 300 medicinal compounds, only six compounds: arzanol, ferulic acid, genistein, resveratrol, rosmanol and thymohydroquinone showed significant interaction with the SARS viral proteins by forming hydrogen bonds with the active site residues with low binding energy. Further ADMET (absorption, distribution, metabolism, excretion and toxicity) analysis showed good pharmacokinetic properties and low acute toxicity of these compounds. **Conclusion:** The current study provides convincing evidence that these medicinal compounds exert antiviral activity against the SARS-COV-2 virus and could be further exploited for the treatment of this disease.

Severe acute respiratory syndrome coronavirus-2 (SARS-COV-2), a novel pathogen from the class of coronaviruses, is taking over the world at an unprecedented rate. It is one of the most debilitating and deadly viral respiratory diseases, and has raised many concerns among healthcare professionals and the general public. The dilemma is the severity profile, enhanced surged capacity of diagnostic and laboratory tools, the spectrum of illness and that there is no specific therapeutic intervention, while the lack of sufficient understanding of viral pathogenesis makes it even more enigmatic [1,2]. Recent studies are currently underway to decipher the molecular mechanism adopted by these viruses to understand the nature of the disease and to identify potential therapeutic targets for vaccine and drug development.

Coronaviruses, by taxonomical hierarchy, belong to order Nidovirales and family Coronaviridae. The family is further genetically classified into four different genera: Alphacoronavirus, Betacoronavirus, Gammacoronavirus and Deltacoronvirus. The first two genera (alpha and beta-) of coronaviridae cause diseases in mammals, whereas the other two affect birds. Both SARS-COV-1 (Middle East respiratory syndrome) and SARS-COV-2 (Novel Coronavirus-19) belong to genus Betacoronavirus that are highly contagious viruses and notorious for causing a multitude of diseases with neurological, respiratory, enteric and hepatic manifestations [3–5]. They are assumed to be transmitted from bats to different organisms, which ultimately lead to transmission to humans.

SARS-COV-2, previously known as Novel coronaviruses (nCOV-19), are enveloped positive-sense single-stranded RNA viruses with a genome size of 27–30 kb and are equipped with 5′-cap structure and 3′ poly-A tails that act as typical messenger RNA upon transduction [6,7]. The genomic organization of SARS-COV-2 is comprised of 14 annotated open-reading frames (ORFs) translating 27 proteins. At the 5′-terminus, lies ORF1a and ORF1b, which encode for polyproteins that after proteolytic processing produce 16 nonstructural proteins (NSP), involved in direct RNA replication and transcription. The structural region is congregated adjacent to the 3′-end and encodes for four structural proteins (spike, envelope, membrane and nucleocapsid) and other accessory proteins through ribosomal frameshifting.

The function of SARS spike glycoprotein is to recognize angiotensin converting enzyme-2 receptor on human epithelial cells to attach and transduce its viral RNA, to commence their proliferation [8], whereas membrane and envelope proteins are responsible for viral assembly and packaging. Nucleocapsid protein encapsulates the viral genetic material inside the virion [9]. Among NSPs, NSP-5 protease [10] and peptidase [11] facilitate polyprotein cleavage, NSP-9 replicase [12] encourages replication of viral RNA and NSP-15 endoribonuclease, [13] (a subunit of SARS-COV-2 RNA-dependent RNA polymerase) catalyzes viral RNA replication. The role of other NSPs is still elusive. All these protein culprits together cause serious respiratory distress sometimes leading to death. Inhibiting some of these viral proteins might impede viral replication and could be considered invaluable therapeutic targets for this disease.

To recognize potential inhibitors of these viral proteins, initial screening of various pharmacological agents is essential. Among these agents, medicinal compounds are promising drug candidates for SARS-COV-2, as they are known for their antiviral and immunomodulatory activity since medieval times and some of them are well studied [14–16]. Other abilities of these compounds include immune response amelioration, rejuvenation of damaged tissues [16,17] and reduction of viral load [15,18]. Here, we analyzed different medicinal compounds using a virtual screening method to obtain promising inhibitors for these viral proteins that could be further utilized for SARS-COV-2 treatment.

## Materials & methods

In this study, essential SARS-COV-2 proteins facilitating viral–host interaction, polyprotein processing and vital replication proteins were selected from the RCSB protein databank. These proteins are SARS coronavirus peptidase (2GTB) [11], SARS-COV-2 Protease (6LU7) [10], Spike Glycoprotein (6VSB) [8], NSP-9 replicase protein (6W4B) [12] and NSP-15 endoribonuclease (6VWW) [13]. ModRefiner was used for structure refinement and energy minimization of these protein receptors [19]. The refined protein structures were used to predict their active site residues using Metapocket 2.0 [20] and subsequently exploited for docking analysis. Medicinal compounds, known for antiviral and the immunomodulatory activity [14–16,18], were selected as ligands and prepared with the PRODRG server [21] prior to docking. The entire docking study was facilitated by Autodock vina equipped with Raccoon2 (a virtual screening plugin), and the ligands were focused at the predicted active site of the viral protein receptors to perform site-specific docking to procure potential inhibitors. The successful ligands obtained from screening analysis were further investigated for ADMET (absorption, distribution, metabolism, excretion and toxicity) properties facilitated by GUSAR [22] and SwissADME database [23].

## Results

### Molecular docking results

**Figure 1.**
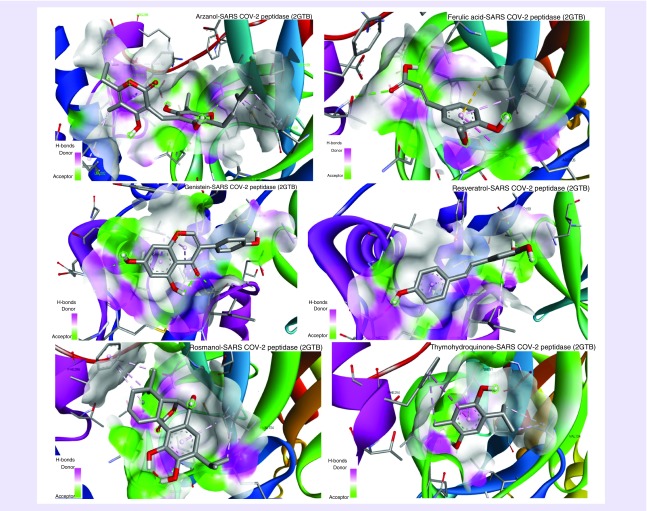
**Molecular view of medicinal compounds interacting with of severe acute respiratory syndrome coronavirus-2 peptidase protein.**

Molecular docking is a computer-assisted drug design and development method utilized to predict the interaction and binding mode of different compounds with a target protein. These compounds (ligands) when focused at the active site of a particular protein (receptor) establish different types of chemical bonds with their active residues. These chemical bonds include hydrogen, Vanderwaal, salt bridges, π–π interaction, π-sigma bond, π-sulfur and many other hydrophobic interactions. Among these bonds, hydrogen bonds play a critical role in protein–ligand interaction and lower the binding energy in order to stabilize the docked complex [24]. It is already established that when a protein active site is blocked by a chemical substrate (ligand) this abrogates its functional enzymatic activity. This approach was adopted to find novel inhibitors for SARS-COV-2 by inhibiting critical proteins responsible for virus attachment and replication. More than 300 medicinal compounds with immunomodulatory and antiviral activity were added to the Raccoon2 plugin of Autodock vina to perform virtual screening to obtain promising inhibitors for SARS-COV-2 proteins. Both target proteins and compounds were prepared for docking studies by subjecting them for energy minimization and structure refinement. Molecular docking was performed by exposing these compounds to the predicted active site residues of these viral proteins. The results obtained from this analysis were filtered based on compounds interaction with the active site residues, low binding energy and hydrogen and hydrophobic interaction. It was observed that, among these medicinal compounds, only six compounds showed promising interaction with the active site of SARS proteins as summarized in Table 1 and molecularly represented in Figure 1 and Supplementary Figures 1, 3, 5 and 7, respectively.

**Table 1.** **Docking interaction of selected medicinal compounds with the predicted active sites of severe acute respiratory syndrome coronavirus-2 proteins.**

Receptors	Ligands	Predicted active site residues	Hydrogen bonds established by receptor residues with ligand	Binding affinity (kcal/mol)	LigRMSD results (Å)
2GTB	Arzanol	LYS102, VAL104, ARG105, ILE106, **GLN107**, PRO108, GLY109, GLN110, **THR111**, GLN127, PRO132, **ASN151**, ILE152, ASP153, ASP155, CYS156, SER158, CYS160, **THR199**, ILE200, LEU202, ASN203, GLU240, PRO241, LEU242, THR243, ASP245, ASP248, ILE249, PRO252, LEU253, THR292, PRO293, PHE294, ASP295, VAL297, ARG298	**THR111, ASN151**	-7.0	1.11
	Ferulic acid		**THR111, ASN151, ASN292**	-5.4	1.47
	Genistein		**THR199, GLY275, LEU287**	-6.6	1.53
	Resveratrol		**THR199, ASP289**	-5.8	1.45
	Rosmanol		**GLN107**	-6.5	1.51
	Thymohydroquinone		**THR111**	-5.0	1.13
6LU7	Arzanol	THR24, THR25, **THR26**, LEU27, HIS41, VAL42, CYS44, THR45, GLU47, MET49, LEU50, ASN51, **PRO52**, TYR54, **ALA70**, **GLY71**, **ASN95**, TYR118, ASN119, PHE140, LEU141, **ASN142**, **GLY143**, SER144, CYS145, HIS163, HIS164, MET165, **GLU166**, LEU167, PRO168, HIS172, ASP187, **ARG188**, **GLN189**, THR190, ALA191, ALA193, GLN192	**GLN189**	-6.3	0.998
	Ferulic acid		**GLU14, MET17, GLY71**	-4.7	1.00
	Genistein		**THR26, ASN142, GLY143, GLU166**	-6.6	0.98
	Resveratrol		**PRO52, ASN180, ARG188**	-5.8	1.04
	Rosmanol		**ASN142, GLN189**	-6.7	1.03
	Thymohydroquinone		**ALA70, ASN95**	-5.0	1.02
6VSB	Arzanol	**ARG319**, CYS391, LEU517, PHE543, ASN544, GLN564, **PRO589**, **SER591**	**ARG319**	-6.5	1.34
	Ferulic acid		**SER591**	-4.8	1.37
	Genistein		**SER591, PRO589**	-6.6	1.04
	Resveratrol		**SER591**	-5.8	1.31
	Rosmanol		**SER591, CYS538**	-6.7	0.35
	Thymohydroquinone		**PRO589, SER591**	-5.0	1.33
6VWW	Arzanol	GLU69, VAL70, **LYS71**, ILE72, TYR89, **LYS90**, PRO158, GLN160, GLY165, VAL166, THR167, LEU168, PHE195, THR196, **GLN197**, SER198, ARG199, ASN200, **LEU201**, GLN202, GLU203, LEU252, LEU266, GLU267, ASP268, PRO271, MET272, ASP273, SER274, THR275, VAL276, **LYS277**, **TYR279**, VAL295, ILE296, ASP297, ASP324	**LYS90, LYS277, TYR279**	-7.0	0.84
	Ferulic acid		**GLN160**	-5.4	1.33
	Genistein		**LYS71, ASN75, SER329**	-6.4	0.70
	Resveratrol		**GLN197**	-6.2	0.61
	Rosmanol		**TYR279**	-6.8	1.17
	Thymohydroquinone		**LEU201**	-5.0	0.98
6W4B	Arzanol	MET13, SER14, CYS15, GLY38, GLY39, **ARG40**, PHE41, **VAL42**, ARG56, PHE57, PRO58, LYS59, **SER60**, ASP61, ILE66, TYR67, THR68, ILE92	**ARG40**	-6.5	1.02
	Ferulic acid		**SER60**	-5.8	1.11
	Genistein		**VAL42**	-6.9	1.23
	Resveratrol		**ARG40, SER60**	-5.8	1.43
	Rosmanol		**SER60**	-6.6	1.39
	Thymohydroquinone		**ARG40, SER60**	-5.2	1.02

RMSD: Root-mean-square deviation.

Bold value represents amino acids residues which are the part of the predicted active site that participated in the hydrogen bonding with the ligands.

**Figure 2.**
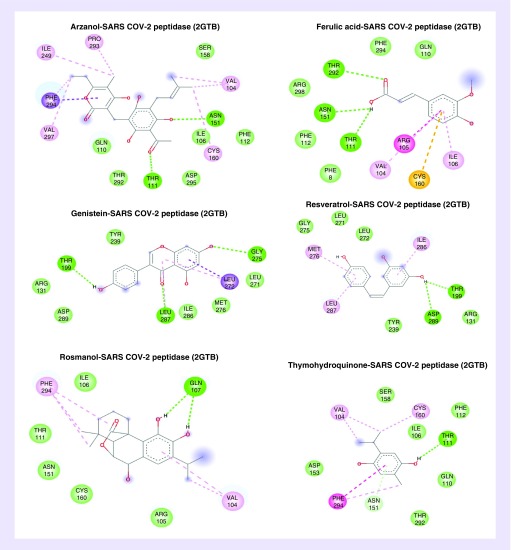
**Structural representation of medicinal compounds forming hydrogen and other miscellaneous bonds with severe acute respiratory syndrome coronavirus-2 peptidase protein.** *Green interaction line depicts hydrogen bond, pink: amide–pi stacking, purple: pi–sigma bond and light yellow: pi–sulfur interaction.

The majority of these compounds exploited oxygen (O-, O=) and hydroxyl groups (-OH) to form hydrogen bonds with the SARS proteins whereas carbon rings participated in establishing hydrophobic interactions. Some compounds showed similar binding affinity for the active residues of these viral proteins to establish hydrogen bonds, such as in the case of peptidases (2GTB), Threonine-111 and Asparagine-151 were favored by Arzanol, Ferulic acid and Thymohydroquinone while Threonine-199 by Genistein, and Resveratrol as shown in Figure 2. For protease (6LU7), compounds like Arzanol, Genistein and Rosmanol preferred Asparagine-142 and Glutamine-189 for interaction (Supplementary Figure 2), whereas in spike glycoprotein (6VSB), all the compounds except Arzanol shared a common residue Serine-591 for bond formation (Supplementary Figure 4). Among nonstructural proteins, Arginine-40, Serine-60 of NSP-9 replicase (6W4B) protein are involved in forming hydrogen bonds with all compounds except for Genistein that has chemical affinity for Valine-42 (Supplementary Figure 6). For NSP-15 endoribonuclease (6VVW), Tyrosine-279 was the common binding residue for Arzanol and Rosmanol (Supplementary Figure 8). The binding energy observed for these compounds was approximately 5–7.0 kcal/mol and they were further subjected for ligand conformational analysis, which was predicted by the LigRMSD database [25]. The prime purpose of root-mean-square deviation (RMSD) analysis is to evaluate experimentally solved ligand structure against predicted docked ligand conformations. The predicted value of the RMSD for these compounds was approximately 0.9–1.5 Å, which is considered successful and stable. A value beyond 2 Å indicates instability and aberrancy in ligand conformation and docking parameters.

### Acute toxicity studies

The principal aim of acute toxicity prediction is to determine unwanted side effects of a compound after single or multiple exposures to an organism by a known administration route (subcutaneous, oral, inhalation, intravenous or intraperitoneal). The successful compounds were further used to evaluate their acute toxicity using GUSAR. GUSAR analyses compounds based on the Quantitative Neighborhoods of Atoms descriptors and Prediction of Activity Spectra for Substances algorithm and correlate the obtained results with SYMYX MDL Toxicity Database and further classify them on the Organisation for Economic Co-operation and Development (OECD) chemical classification manual [22]. It has been observed that these compounds elicit toxicity when the compound dose surpasses more than 500,000 mg/kg in case of the oral route, more than 7000 mg/kg for intravenous route and more than 20,000 mg/kg for subcutaneous and intraperitoneal route database as shown in Table 2. The results of the OECD chemical classification of these compounds revealed that Arzanol, Genistein, Rosmanol and Thymohydroquinone are Class 4 chemicals, whereas ferulic acid and resveratrol are Class 5 chemicals.

**Table 2.** ***In silco* prediction of acute toxicity in rodent models and chemical classification of selected medicinal compounds.
**

No.	Ligands	Rat oral LD50 (mg/kg)	Rat IV LD50 (mg/kg)	Rat SC LD50 (mg/kg)	Rat IP LD50 (mg/kg)	OECD chemical classification
1	Arzanol	606,400	95,730	745,600	268,800	Class 4
2	Ferulic acid	2754,000	224,400	1058,000	682,300	Class 5
3	Genistein	1749,000	297,100	2886,000	1196,000	Class 4
4	Resveratrol	2845,000	136,600	1833,000	838,600	Class 5
5	Rosmanol	340,900	7,158	24,920	381,800	Class 4
6	Thymohydroquinone	838,600	81,660	471,700	431,600	Class 4

IP: Intraperitoneal; IV: Intravenous; LD50: Lethal dosage-50; OECD: Organisation for economic co-operation and development; SC: Subcutaneous.

### Pharmacokinetic properties

To ascertain the behavior of these compounds inside an organism in terms of absorption, distribution, metabolism, and excretion (ADME), it is imperative to discover their pharmacokinetic properties prior to animal and clinical studies. For this reason, the SwissADME database [23] was exploited to elucidate the pharmacokinetics and drug likeness of these compounds. The lipophilicity of arzanol had a LogP_O/W_ value of 3.42 that indicates high sublingual absorption, whereas for other compounds the value remained in between 1.3 and 2.8 suggesting high oral, intestinal and central nervous system absorption. All these compounds possess high gastrointestinal absorption and water-soluble capability and ferulic acid, resveratrol and thymohydroquinone are permeable to the blood–brain barrier. Out of six compounds, two of them; ferulic acid and resveratrol are CYP1A2 inhibitors, which increases the drug half-life of these compounds and also avert serious drug interactions. The drug-likeness criteria are qualified by all these compounds with no violations and possess an appreciable bioavailability score. The results are summarized in Table 3.

**Table 3.** **Pharmacokinetic parameters of selected medicinal compounds.**

No.	Ligands	Log P_o/w_ (Lipophilicity)	GI absorption	BBB permeant	CYP1A2 inhibitor	Druglikness (Lipinski, violations)	Bioavailability score	Water solubility (Log S)
1	Arzanol	3.42	High	No	No	Yes; 0 violation	0.55	-4.70 (Moderately Soluble)
2	Ferulic acid	1.36	High	Yes	No	Yes; 0 violation	0.56	-2.11 (Soluble)
3	Genistein	2.04	High	No	Yes	Yes; 0 violation	0.55	-3.72 (Soluble)
4	Resveratrol	2.48	High	Yes	Yes	Yes; 0 violation	0.55	-3.62 (Soluble)
5	Rosmanol	2.88	High	No	No	Yes; 0 violation	0.55	-4.25 (Moderately Soluble)
6	Thymohydroquinone	2.39	High	Yes	Yes	Yes; 0 violation	0.55	-3.03 (Soluble)

BBB: Blood–brain barrier; CYP: Cytochromes P450; GI: Gastrointestinal tract.

## Discussion

With a growing number of infected cases with thousands of mortalities worldwide, it is imperative to discover potential therapies in order to curb this devastating situation. WHO approved chloroquine on an emergency basis and hydroxychloroquine based on promising *in vitro* and clinical results [26]. But in hindsight, these drugs are highly toxic, capable of jeopardizing patient’s health by disrupting essential heart and neurological functions [27,28]. On the other hand, hundreds of different anti-HIV protease inhibitors have been reported that are capable of terminating SARS-COV-2 viral replication and RNA extension. However, these viruses possess proof-reading and other coping mechanisms to attenuate the interaction, thus making these antiprotease drugs inefficient as evident from recent clinical trials [9,28,29]. To address these problems, we utilized a novel approach by gathering medicinal compounds with antiviral and immunomodulatory activity to check their inhibitory interaction against critical SARS-COV-2 proteins. The viral proteins include structural proteins such as spike glycoprotein that allow these viral particles to attach themselves to ACE-2 receptor of the host cell, whereas nonstructural proteins (NSP-9, NSP-15) facilitate viral replication and proteases modulate the production of different proteins through proteolytic cleavage of SARS-COV-2 polyproteins. This study aimed to obtain novel drug candidates that have the capability to interact with the active site of all of these viral proteins and should possess efficient pharmacokinetic profile with low toxicity to ensure safety during administration.

Both proteins and ligands were prepared with Modrefiner and PRODRG server to eradicate any bad contacts, unwanted potential energy and structural anomaly that might result in false interaction. These structurally refined molecules were used and added to raccoon2 virtual screening plugin of Autodock vina to perform virtual screening of these ligands and to filter out possible drug candidates establishing hydrogen bonds, other than Vanderwaal interaction, with the active site residues of these viral protein receptors. Only six medicinal compounds: arzanol, ferulic acid, genistein, resveratrol, rosmanol and thymohydroquinone formed hydrogen bonds with these viral proteins. These compounds were further analyzed for ligand RMSD to evaluate their interaction stability through LigRMSD [25]. The predicted value of RMSD of these compounds was in between 0.9–1.5 Å, which is considered adequate and stable [30].

Determining the acute toxicity and pharmacokinetic properties of these compounds was facilitated by GUSAR software [22] and SwissADME [23]. The toxicity profile of these compounds is relatively low and require high doses to elicit toxic response. The majority of the compounds are Class 4 chemicals that have mild toxic effects (piloerection and diarrhea), whereas ferulic acid and resveratrol are Class 5 chemicals with low toxic effects [31]. Hence, the dosage of these compounds must be calibrated to exploit its full benefits and avert adverse effects. The pharmacokinetic attributes are in favor of these compounds to be exploited as promising drug candidate for SARS-COV-2 treatment.

Apart from their antiviral activity, these medicinal compounds rejuvenate immunological response, and prevent the onset of cytokine storm that is classical hallmark of this disease. Medicinal compounds combined with standard antiviral medications, synergistically ameliorate the inhibitory action on antiviral proteins [32], reduce toxic effects [33] promote tissue repair and alleviate patients’ symptoms [16]. Incorporating these compounds with temporary approved anti-SARS drugs as adjuvants might develop and sustain good immunological response against this lethal infection. Another probable action of these compounds is to encourage phagocytotic functions, regulate the proliferation of macrophages and neutrophils and expedite the development of adaptive immunity by promoting T-cells cytokine production, natural killer cells activity and dendritic cells stimulation, which in reality takes 4–7 days for activation [14,16]. Such intervention might be able to build immunity and ameliorate the deplorable condition of SARS-COV-2-affected individuals.

## Conclusion

Virtual screening of different immunomodulatory compounds successfully filtered promising drug candidates for the treatment of SARS-COV-2. These compounds have low toxicity, efficient pharmacokinetic profile and an excellent binding affinity for SARS-COV-2 proteins. They may be further subjected to *in vitro* and *in vivo* experimentations to determine their therapeutic efficacy to be further exploited as lead compounds for clinical trials. This study elucidated the chemical affinity and mechanism of these medicinal compounds against SARS-COV-2 protein targets. The results obtained from this *in silico* study could be used as a vehicle by other researchers and clinicians looking for promising anti-SARS therapies.

**Summary points**

- Docking interaction of immunomodulatory medicinal compounds library filtered six promising medicinal compounds against severe acute respiratory syndrome coronavirus-2 (SARS-COV-2) viral proteins.
- These six compounds, arzanol, ferulic acid, genistein, resveratrol, rosmanol and thymohydroquinone, possess strong binding affinity for SARS-COV-2 proteins by forming hydrogen bonds within active site residues of these proteins.
- These compounds have low acute toxicity profile, excellent pharmacokinetic properties and show nominal deviation from the viral protein backbone, as evident from their root-mean-square deviation values.
- This study suggests that these compounds are promising anti-SARS-COV-2 inhibitors and can also serve as immunomodulatory agents to enhance immunogenicity.
- The findings of this study might serve as a guide for clinicians, pharmaceutical experts and other researchers to further validate these insights *in vitro* and in large clinical settings to confirm their therapeutic efficacy in this disease.
